# AAV-CapNet: Multi-Omics Deep Learning with Fourier Enhancement and Cross-Attention for Predicting Infectivity of Known AAV Capsid Variants

**DOI:** 10.3390/v18101093

**Published:** 2026-10-03

**Authors:** Junhan Dong, Zhiyun Cheng, Yong Diao

**Affiliations:** School of Medicine, Huaqiao University, Quanzhou 362021, China; chengzhiyun@hqu.edu.cn

**Keywords:** AAV, gene therapy, multi-omics integration, deep learning, infectivity prediction

## Abstract

This work builds AAV-CapNet, a deep learning model integrating Fourier frequency enhancement and cross-attention fusion, to predict the infectivity of 20 AAV capsid variants across 52 cancer cell lines with a self-built multi-omics dataset of 392 validated measurements. Validated via stratified splitting and repeated five-fold cross-validation, it extracts dual-domain capsid features fused with host transcriptomics for joint classification and regression. The model exhibits favourable performance under low-false-positive-rate conditions suitable for high-throughput screening. Attention mapping and ablation tests validate core modules’ necessity. Despite poor generalization to unseen serotypes, this interpretable tool facilitates intraserotype capsid screening and rational engineering.

## 1. Introduction

Adeno-associated virus (AAV) is the leading vector for in vivo gene delivery due to its non-pathogenicity, low immunogenicity, and sustained transgene expression. Approved AAV therapies include Luxturna, Zolgensma, and Elevidys, approved by the US Food and Drug Administration (FDA) [1,2]. Efficacy depends on capsid infectivity and tissue tropism encoded by the cap gene, with genomic variations altering receptor binding, entry, and immunogenicity [2,3,4,5]. AAV transduction is a reciprocal virus–host process in which capsid sequences define surface motifs and host transcriptomic profiles determine receptor and endocytic machinery abundance [1,3]. Predicting infectivity therefore requires modeling host–virus interactions simultaneously, as capsid sequences alone are insufficient to account for host-driven differences across cell lines.

High-throughput screening of AAV capsid libraries via cell-based infectivity assays remains prohibitively expensive and time-consuming, motivating the development of computational methods for predicting sequence–function relationships [1,3,6]. Early predictive frameworks for AAV capsids fall into three categories with separate limitations. Sequence-only models such as Random Forest, XGBoost, CNN and LSTM take only capsid amino acid sequences and miss cell-type-specific transduction differences due to receptor expression variation [4,7,8]. Generative design models such as VAEProp and AAVGen focus on generating novel sequences rather than predicting infectivity across cell lines and lack integration of host transcriptomic data, and offer limited interpretability of functional residues [9,10,11,12]. Preliminary host-aware pipelines incorporate some cell expression data but use simple concatenation fusion without dynamic cross-alignment between viral and host features, resulting in suboptimal regression accuracy for continuous infectivity values.

Most sequence-only deep learning models for AAV capsids share three well-documented limitations. First, CNN and LSTM encoders extract only local temporal features, overlooking global periodic spectral patterns that reflect evolutionarily conserved structural periodicity in capsid proteins [13,14]. Second, they omit host transcriptomic signatures of receptor and endocytic genes—a critical gap, as host expression drives cell-specific infectivity variance. Third, these models operate as uninterpretable black boxes without residue-level attribution tools, making it impossible to map predictions to functional capsid domains such as HVRs or RBDs.

AAV-CapNet integrates AAV capsid sequences with host transcriptomic and receptor expression data. It employs DFT-based frequency-domain enhancement to capture long-range periodic structural signals, including symmetric hypervariable loops and conserved β-barrel cores overlooked by standard encoders, complementing local CNN-derived motifs. A cross-attention fusion module dynamically aligns viral and host multi-omics features, enabling simultaneous binary infectivity classification and continuous infectivity regression. Evaluated on a self-constructed multi-omics dataset, the model advances high-throughput AAV screening and engineering via four core innovations. It integrates a Fourier-enhanced sequence encoder to capture evolutionarily conserved structural constraints, adopts dynamic cross-attention to outperform static concatenation when modeling cell-line-specific tropism across diverse cellular contexts, leverages a weighted joint classification–regression loss framework to balance rapid identification and accurate quantification of transduction activity for ranking, and implements unsupervised interpretable attention mapping to pinpoint functionally critical receptor-binding and hypervariable residues. This design directly guides rational capsid mutagenesis and overcomes the inherent black-box limitations of conventional models.

## 2. Materials and Methods

### 2.1. Multi-Omics Dataset Construction

AAV-CapNet was trained on a curated multi-omics dataset. AAV capsid sequences (encompassing 14 serotype backbones and engineered variants) were retrieved from the AAV Registry and the published literature, whereas host datasets derived from 52 distinct immortalized cancer cell lines, including canonical models such as HepG2 and HEK293T, were acquired from the Cancer Cell-Line Encyclopedia (CCLE), a database of immortalized cancer cell lines [8,15,16]. For each cell line, 16 core transcriptomic features were extracted, encompassing the primary receptor AAVR, key co-receptors, heparan sulphate biosynthesis genes, endocytosis regulators, and known transduction modulators. Infectivity measurements were aggregated from both public repositories (DeepAAV, AAV Tracker) and in-house assays, standardized to transgene fluorescence enrichment relative to mock controls [8,15].

Strict quality control measures were implemented throughout data processing. The analysis exclusively retained full-length cap sequences and cell lines featuring complete marker profiles. For all quantitative measurements, no fewer than two biological replicates were collected, signals from three technical replicates were averaged, and data entries with elevated coefficients of variation or outliers identified via interquartile range filtering were discarded. Post-filtering, 392 validated records remained. Data harmonization included $\log_{2}$ transformation of infectivity values, global median centering and batch-wise variance scaling, and z-score normalization of transcriptomic features.

The dataset was stratified by serotype and cell line into training, validation, and test sets to prevent data leakage, with five-fold cross-validation confined to the training subset. Classification thresholds were determined via a median-based split to ensure balanced labels, with robustness verified against quartile cutoffs. To address the limited sample size, the model utilized lightweight architectures, feature compression, and comprehensive regularization (dropout, weight decay, gradient clipping, early stopping), ensuring stable performance suitable for small-sample deep learning tasks.

### 2.2. Multi-Dimensional Sequence Encoding

Each AAV capsid amino acid sequence of length *L* was transformed into an intermediate concatenated feature matrix $X_{\mathrm{inter}}\in R^{L\times 97}$ by concatenating five groups of feature representations. This 97-dimensional intermediate matrix is projected via a learnable linear layer down to $X_{\mathrm{seq}}\in R^{L\times 64}$.

One-hot encoding spans 21 dimensions, covering the 20 standard amino acids along with a padding gap symbol denoted by a dash. Each residue is encoded as a binary vector of length 21, containing a single value of one at the index corresponding to that amino acid and zeros in all remaining positions. This yields a discrete and orthogonal representation that preserves mutation information while avoiding unintended ordinal assumptions.

Physicochemical properties spanning seven dimensions were extracted from the AAindex database for each amino acid [13]. This included hydrophobicity based on the Kyte-Doolittle scale, polarity according to Grantham’s polarity measure, net charge at pH 7, molecular weight measured in Daltons, hydrogen bond donor and acceptor propensity, aromaticity distinguishing tyrosine, phenylalanine and tryptophan from other residues, and flexibility indicated by proline or glycine propensity. Collectively, these properties directly influence capsid stability, receptor binding, and membrane interaction.

Positional encoding comprises 64 dimensions, matching the final feature dimension. Following the Transformer architecture, sinusoidal positional encoding is applied to embed sequential positional information, as specified in Equation (1) [17].

(1) $$PE_{\left(\mathrm{pos},2i\right)}=\sin\left(\frac{\mathrm{pos}}{{10,000}^{\frac{2i}{d}}}\right),\;PE_{\left(\mathrm{pos},2i+1\right)}=\cos\left(\frac{\mathrm{pos}}{{10,000}^{\frac{2i}{d}}}\right)$$

where pos represents the positional index of amino acid residues along the capsid protein sequence, *i* denotes the feature dimension index, and *d* is fixed at 64 as the constant output dimension of the positional encoding module.

Local window features span three dimensions. For each residue *i*, we adopted a window of size five with two residues flanking each side, or a smaller window close to the protein termini. We further computed three summary statistics, including the fraction of hydrophobic residues in the window, the fraction of positively and negatively charged residues, and the fraction of polar uncharged residues. This approach captures short-range physicochemical context.

Global sequence features span two dimensions. The first is the cumulative average hydrophobicity from position one to position *i* normalized by the total sequence length, and the second is the relative position calculated as *i* divided by *L*. These features capture the global hydrophobicity gradient along the capsid protein chain, which is known to influence protein folding and stability. The five feature groups are concatenated into a 97-dimensional intermediate vector consisting of 21 dimensions from one-hot encoding, seven dimensions from physicochemical properties, 64 dimensions from positional encoding, three dimensions from local window features, and two dimensions from global sequence features. A learnable linear projection layer maps this 97-dimensional representation to 64 dimensions to form the final low-dim sequence representation $X_{\mathrm{seq}}\in R^{L\times 64}$.

The full set of 392 validated records is split into training, validation, and test subsets via stratified sampling by serotype and cell line. The training set accounts for 60% of the total dataset and contains 235 records. The validation set accounts for 20% and contains 78 records. The test set corresponds to the remaining 20% and contains 79 records. All five-fold cross-validation runs are conducted exclusively within the training subset to avoid data leakage.

### 2.3. Full Tensor Dimension Flow

We report complete tensor shape progression from raw amino acid sequence to cross-attention inputs. Starting from a raw amino acid sequence of length *L*, concatenated multi-source hand-crafted features yield the intermediate representation $X_{\mathrm{inter}}\in R^{L\times 97}$. A subsequent linear projection transforms this intermediate tensor to obtain $X_{\mathrm{seq}}\in R^{L\times 64}$. This tensor serves as input for the 1D-CNN module and the module produces $F_{\mathrm{conv}}\in R^{L\times 64}$ after feature extraction, and feature maps are interpolated back to original sequence length *L* following pooling operations. The tensor $F_{\mathrm{conv}}$ is then fed into the Bi-LSTM module. The bidirectional structure delivers $H_{\mathrm{lstm}}\in R^{L\times 256}$ through concatenation of outputs generated from 128 hidden units per direction.

Next comes DFT frequency-domain enhancement. Discrete Fourier transform operates on each channel of $H_{\mathrm{lstm}}$ and magnitude spectrum is retained for further processing. Per-channel standard normalization is conducted on the magnitude spectrum, and normalized outputs are interpolated back to length *L* to form $F_{\mathrm{fft}}\in R^{L\times 256}$. We then combine $H_{\mathrm{lstm}}$ and $F_{\mathrm{fft}}$ along the feature dimension to reach a dimension of 512. The combined representation flows through a fully connected layer that maps 512 dimensions down to 256 dimensions so that we can acquire the final sequence feature $F_{\mathrm{seq}}\in R^{L\times 256}$.

For cross-attention computation, host-derived feature tensor $F_{\mathrm{cell}}\in R^{N\times 16}$ works together with sequence feature $F_{\mathrm{seq}}\in R^{L\times 256}$. Corresponding projection matrices are $W_{q}\in R^{16\times 32}$, $W_{k}\in R^{256\times 32}$ and $W_{v}\in R^{256\times 32}$.

### 2.4. Fourier-Enhanced Sequence Encoder

The sequence encoder comprises three cascaded modules. These include a one-dimensional CNN for local motif extraction, a bidirectional LSTM for global context modeling, and a discrete Fourier transform module for frequency-domain feature enhancement.

The 1D CNN module consists of two convolutional layers with a kernel size of 3, stride of 1, “same” padding, and ReLU activation. The first layer accepts 64 input channels and outputs 32 channels, followed by a MaxPool1d layer with pool size 2 and stride 2. The second layer maps 32 input channels to 64 output channels, followed by another MaxPool1d layer with pool size 2 and stride 2. The pooled feature map is upsampled back to the original sequence length L using linear interpolation to preserve positional alignment with the input sequence. The resulting $F_{\mathrm{conv}}\in R^{L\times 64}$ captures local amino acid motifs such as the conserved RGD or GXXG motifs critical for receptor recognition.

The bidirectional LSTM module comprises one layer with 128 hidden units in each direction, yielding a total of 256 output dimensions per time step, and takes $F_{\mathrm{conv}}$ as input. The forward LSTM processes the sequence from left to right to capture dependencies spanning the N-terminus to the C-terminus, while the backward LSTM reads the sequence in the reverse right-to-left order. The output hidden states from the forward and backward passes are concatenated at each sequence position, producing $H_{\mathrm{lstm}}\in R^{L\times 256}$. This module captures long-range interactions, such as the coupling between the N-terminal phospholipase A2 domain and the C-terminal VP3 region.

The DFT frequency module applies the discrete Fourier transform independently to each of the 256 feature channels of $H_{\mathrm{lstm}}$. For a given channel h(t) with *t* = 0 to L−1, the DFT is as defined in Equation (2) [14,18].

(2) $${\hat{h}}_{k}=\sum_{t=0}^{L-1}h\left(t\right)\cdot e^{-j\frac{2\pi }{L}kt},\;k=0,1,\ldots ,L-1$$

where $|{\hat{h}}_{k}|\in R^{L}$ represents the frequency-domain magnitude spectrum of a single feature channel, *L* denotes the total length of the capsid amino acid sequence, *k* stands for the frequency index, and *j* refers to the imaginary unit applied in the discrete Fourier transform. The magnitude spectrum $|{\hat{h}}_{k}|$ is extracted and normalized to zero mean and unit variance along the frequency axis *k*, and then directly adopted as frequency-domain features. To preserve the original sequence length *L*, the normalized magnitude spectrum is linearly interpolated back to *L* positions. The resulting frequency features $F_{\mathrm{fft}}\in R^{L\times 256}$ are concatenated with the time-domain features. This operation extracts periodic patterns that reflect the structural periodicity of the AAV β-barrel core and the surface loop regions, capturing evolutionarily conserved features that contribute to capsid folding stability and receptor interaction interfaces [19]. The output $F_{\mathrm{fft}}\in R^{L\times 256}$ is concatenated with $H_{\mathrm{lstm}}$ along the channel dimension and passed through a fully connected layer (512 to 256) to produce the final genomic encoding $F_{\mathrm{seq}}\in R^{L\times 256}$.

### 2.5. Host Multi-Omics Encoder and Cross-Attention Fusion

The host feature encoder takes the standardized cell features $X_{\mathrm{cell}}\in R^{N\times 16}$ (with *N* the number of samples) and passes them through two fully connected layers with ReLU activation and dropout (rate 0.3). The first layer expands the dimension to 32, and the second layer compresses back to 16. This architecture captures nonlinear interactions among the 16 input features, for example, synergy between AAVR and EXT1 expression. The output is $F_{\mathrm{cell}}\in R^{N\times 16}$.

Cross-attention uses $F_{\mathrm{cell}}$ as the query *Q* and $F_{\mathrm{seq}}$ as the key *K* and value *V* [11,12]. Dimension alignment via linear projections is defined in Equation (3).

(3) $$Q=W_{q}F_{\mathrm{cell}}\in R^{N\times 32},\;K=W_{k}F_{\mathrm{seq}}\in R^{L\times 32},\;V=W_{v}F_{\mathrm{seq}}\in R^{L\times 32}$$

where $W_{q}\in R^{16\times 32}$, $W_{k}$ and $W_{v}\in R^{256\times 32}$ are learnable weight matrices, $F_{\mathrm{cell}}$ denotes the encoded host cell transcriptomic feature vector, $F_{\mathrm{seq}}$ represents the Fourier-enhanced capsid sequence feature matrix, and $W_{Q}$, $W_{K}$ and $W_{V}$ refer to learnable linear projection weight matrices for the query, key and value branches.

The attention scores and weights are defined in Equations (4) and (5).

(4) $$\mathrm{Score}=\frac{QK^{T}}{\sqrt{32}}\in R^{N\times L},\;{\mathrm{Attn}}_{\mathrm{weight}}=\mathrm{SoftMax}\left(\mathrm{Score}\right)\in R^{N\times L}$$

(5) $$F_{\mathrm{fusion}}={\mathrm{Attn}}_{\mathrm{weight}}\cdot V\in R^{N\times 32}$$

where *Q* and *K* represent the projected query and key feature matrices, respectively, Score denotes the unscaled dot-product similarity matrix from Equation (4), and SoftMax(·) refers to the row-wise SoftMax function that normalizes attention weights into a probability distribution. Division by $\sqrt{32}$ (the scaling factor $d_{k}$) prevents dot products from growing too large, which would drive SoftMax gradients into saturation. The resulting fusion feature $F_{\mathrm{fusion}}$ represents for each cell line a weighted combination of sequence positions, where the weights are determined by how well the host cell’s transcriptomic profile matches the sequence features.

In the ablation variant without cross-attention (w/o Cross-Attention), we replace the cross-attention module with global average pooling over the sequence length dimension (followed by a batch normalization layer with adjusted dimensions) to ensure a fair comparison with a similar number of parameters.

### 2.6. Joint Classification–Regression Output and Optimization

The fusion feature $F_{\mathrm{fusion}}$ is passed through batch normalization, dropout (0.3), and two separate output heads. The classification head is a fully connected layer (32 to 1) followed by Sigmoid activation, producing $P_{\mathrm{cls}}\in \left\{0,1\right\}$ as the predicted probability of high infectivity. The regression head is a fully connected layer (32 to 1) without activation, producing $P_{\mathrm{reg}}\in R$ as the predicted $\log_{2}$ infectivity value.

The model is trained using a weighted joint loss function defined in Equations (6)–(8).

(6) $$L_{\mathrm{total}}=\alpha L_{\mathrm{BCE}}+\left(1-\alpha \right)L_{\mathrm{MSE}}$$

(7) $$L_{\mathrm{BCE}}=-\frac{1}{N}\sum_{i=1}^{N}\left[y_{\mathrm{cls}}^{i}\log P_{\mathrm{cls}}^{i}+\left(1-y_{\mathrm{cls}}^{i}\right)\log\left(1-P_{\mathrm{cls}}^{i}\right)\right]$$

(8) $$L_{\mathrm{MSE}}=\frac{1}{N}\sum_{i=1}^{N}{\left(y_{\mathrm{reg}}^{i}-P_{\mathrm{reg}}^{i}\right)}^{2}$$

where *N* denotes the total number of training samples. $y_{\mathrm{cls}}^{i}$ is the ground-truth binary infectivity label, and $P_{\mathrm{cls}}^{i}$ is the model-predicted probability of high infectivity from the classification head. $y_{\mathrm{reg}}^{i}$ represents the ground-truth normalized continuous $\log_{2}$-transformed infectivity value, while $P_{\mathrm{reg}}^{i}$ indicates the corresponding continuous infectivity output from the regression head. The loss weight coefficient α=0.8 was determined via grid search on the validation set. This weighting scheme places greater emphasis on the classification task, consistent with our primary goal of identifying high-infectivity candidates for gene therapy, while retaining regression supervision to predict the exact quantitative infectivity level.

We employed the Adam optimizer with a set initial learning rate of $10^{-3}$, betas of 0.9 and 0.999 and a fixed weight decay $10^{-5}$. A cosine annealing learning rate scheduler reduces the learning rate to $10^{-5}$ over 50 epochs. Early stopping was applied to monitor validation AUC and training was halted if no improvement was observed for 10 consecutive epochs, and the model weights from the best epoch are restored. Training took approximately 15 min for 20 epochs. The model was implemented in PyTorch 2.0. The lightweight encoder design and cross-attention feature compression greatly reduce the total trainable parameter count, making the model compatible with our limited 392-record dataset. All hyperparameters were determined via grid search over predefined search ranges including initial learning rates of $10^{-4}$, $5\times 10^{-4}$, and $10^{-3}$, dropout rates {0.2,0.3,0.4}, Bi-LSTM hidden dimensions {64,128}, and joint loss weights α∈{x∣0.5≤x≤0.9}. The final optimized model selected a dropout rate of 0.3. The optimal hyperparameter combination was selected solely based on validation-set AUC, without any evaluation or test-set data leakage during tuning. To guarantee full experimental reproducibility, fixed random seeds (seed = 42) were applied uniformly for dataset stratified splitting, model weight initialization, and all repeated five-fold cross-validation replicates.

To eliminate cross-split information leakage caused by mixing identical variants into training and test subsets, we additionally adopted Stratified Group K-Fold cross-validation, with fold-wise quantitative metrics summarized in Appendix A.5 Table A3.

**Figure 1:**
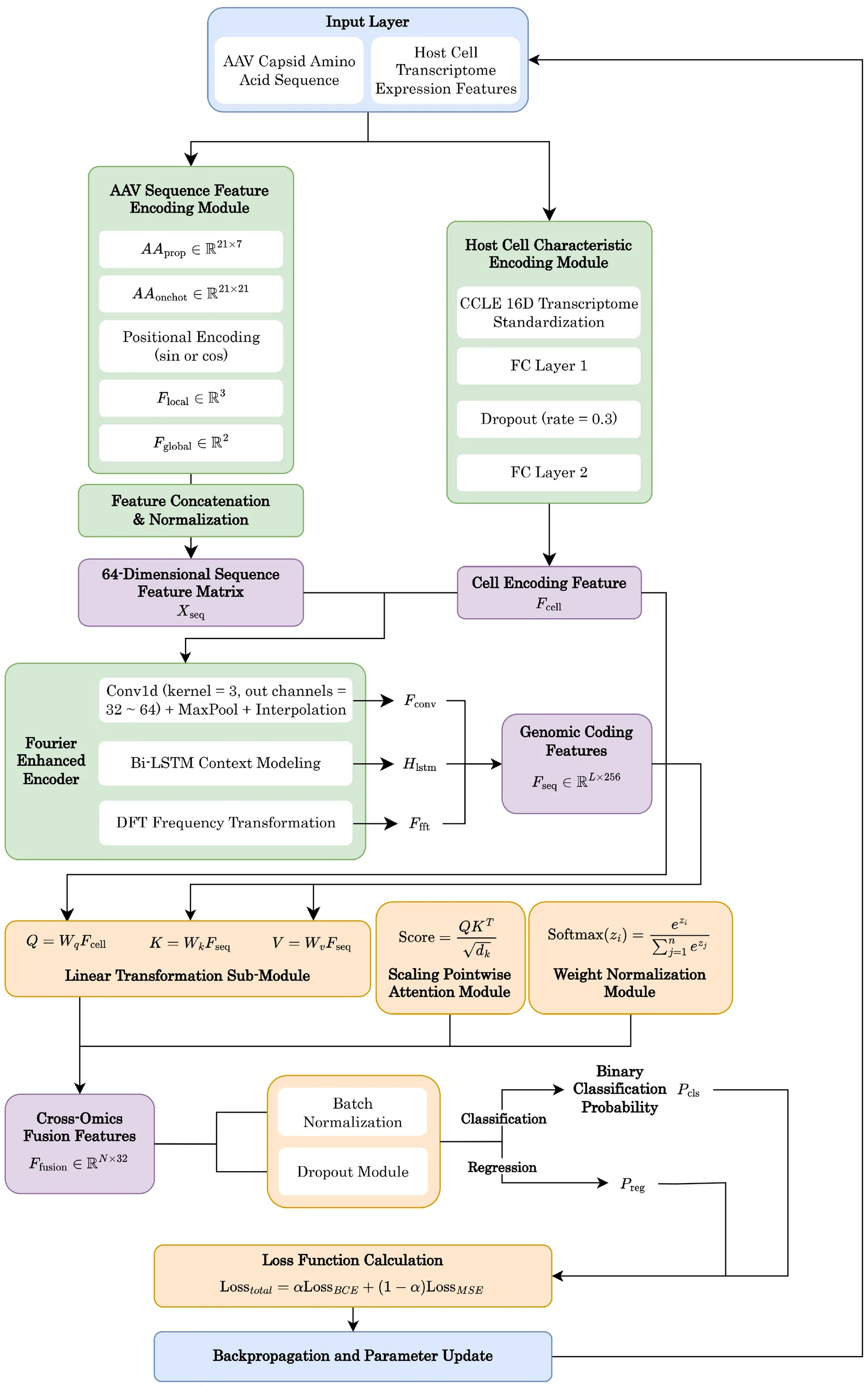
Schematic overview of AAV-CapNet. Input: AAV capsid amino acid sequence (**top**) and host cell CCLE transcriptomic features (**bottom**). Sequence encoding block: 1D CNN (local motifs) to Bi-LSTM (global context) to Fourier transform (frequency-domain periodic features). Host encoder: two fully connected layers with dropout. Cross-attention fusion: host features as query, sequence features as key and value. Joint output heads: classification (Sigmoid) and regression (Linear). The model is trained with weighted BCE plus MSE loss.

A schematic overview of the entire AAV-CapNet architecture is provided in Figure 1, which illustrates the data flow from multi-omics input to joint prediction output.

Additional details on data preprocessing, CCLE feature selection, baseline model configurations, and hyperparameter tuning are provided in the Appendix A.

## 3. Results

### 3.1. Benchmark Comparison Against Baseline Models

**Figure 2:**
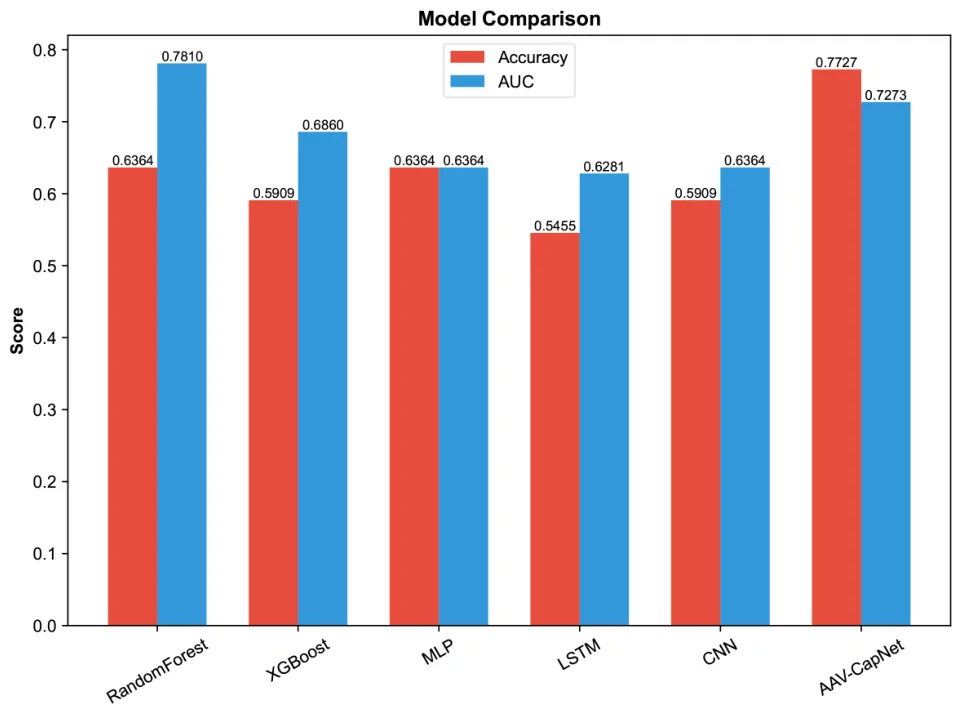
Benchmark performance comparison. Bar plot of accuracy and AUC for all models. AAV-CapNet outperforms baselines in four of five metrics.

AAV-CapNet was benchmarked against five baselines including Random Forest (RF), XGBoost, MLP, LSTM, 1D-CNN under identical data splits and optimized hyperparameters, with the comparison visualized as a bar chart in Figure 2 [4,7,20]. While RF achieved a slightly higher overall AUC (0.7810 vs. 0.7273), AAV-CapNet attained superior accuracy (0.7727), precision (0.8000), recall (0.7273), and $F_{1}$ score (0.7619). Crucially, at false-positive rates below 0.2 (critical for wet-lab screening), AAV-CapNet maintained a true-positive rate over 0.7, outperforming RF, which achieved a true-positive rate below 0.6. Baselines like LSTM and CNN underperformed due to their inability to integrate host multi-omics and frequency-domain features. Repeated 3 × 5-fold cross-validation confirmed that AAV-CapNet achieved a significantly higher mean accuracy of 0.7727, versus 0.6364 for the RF model. This performance gap is statistically supported by a paired *t*-test, which yields a *p* value of 0.003. Rigorous stratified group k-fold results are presented in Appendix A.5 Table A3. This validation design prevents intra-variant information leakage and provides unbiased variant-level performance metrics.

**Figure 3:**
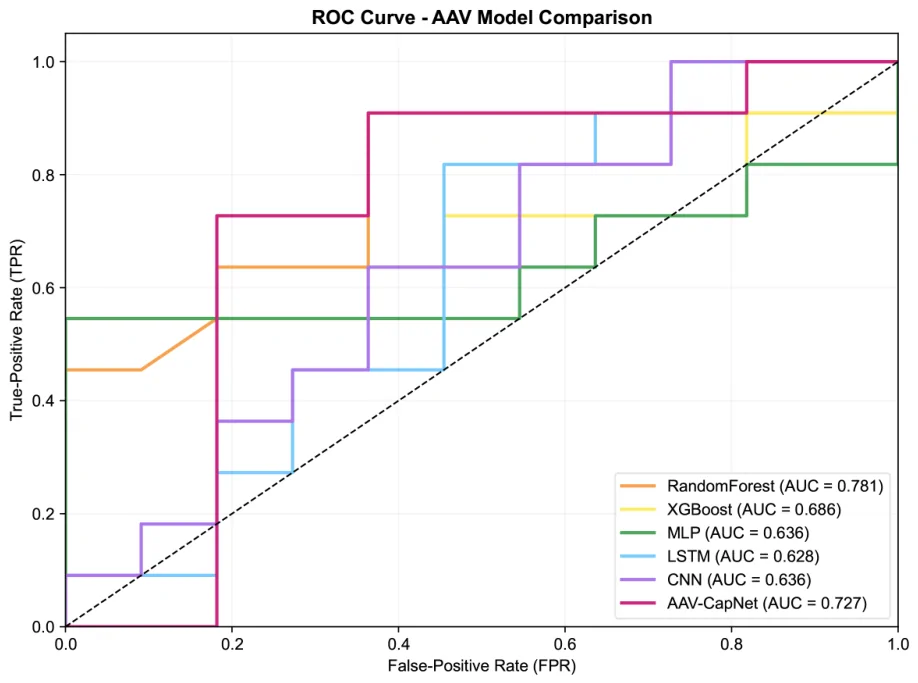
ROC curves. False-positive rate (FPR) versus true-positive rate (TPR). AAV-CapNet (solid red) shows a favourable trade off, especially at low FPR (0 to 0.2), where its TPR exceeds 0.7 while RF and XGBoost drop below 0.6. The dashed diagonal line represents the random classifier baseline, where true positive rate equals false positive rate.

The ROC curves for all models are displayed in Figure 3. AAV-CapNet (solid red) maintains a true-positive rate above 0.7 across false-positive rates from 0 to 0.4, a desirable property for clinical screening where minimizing false positives is critical to avoid costly experimental validation of ineffective vectors. RF has a slightly higher overall AUC (0.781) but its curve dips below AAV-CapNet at low-false-positive-rate regions. The LSTM and CNN curves stay close to the diagonal with AUCs of 0.628 and 0.636 respectively, indicating near random performance. The ROC curves of LSTM and CNN lie close to the diagonal, yielding AUC values of 0.628 and 0.636, which represent only a modest improvement over random prediction.

**Figure A1:**
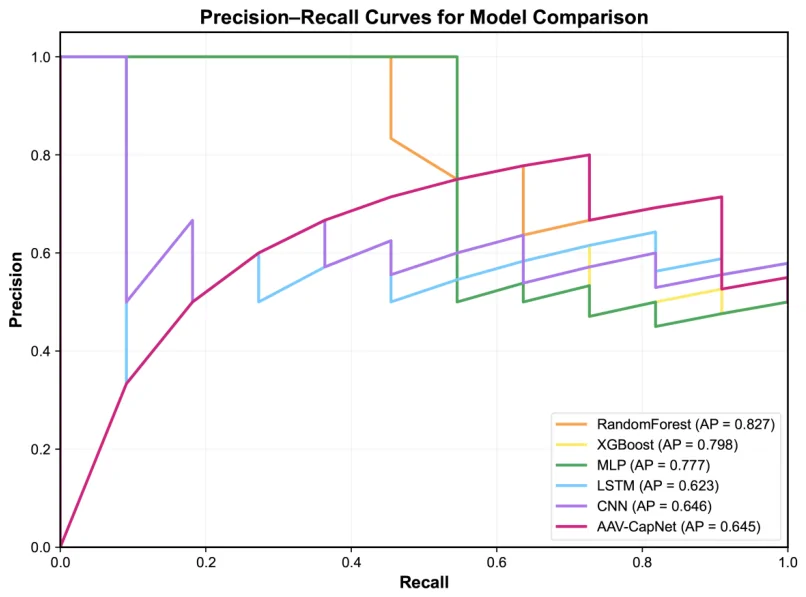
Precision–recall (PR) curves for model comparison. Each curve corresponds to one benchmark model. Average precision (AP) values for individual models are annotated in the legend. PR analysis highlights model performance under class-imbalanced conditions and complements the ROC-based evaluation shown in Figure 3.

To further evaluate model performance under class-imbalanced conditions, precision–recall (PR) curves and average precision (AP) were computed for all benchmark models (Appendix A.1 Figure A1). Random Forest achieved the highest AP of 0.827, followed by XGBoost (AP = 0.798) and MLP (AP = 0.777). By comparison, AAV-CapNet achieved an AP of 0.645. Although Random Forest yielded superior global average precision across all operating thresholds, AAV-CapNet offers a practical advantage for high-throughput screening by maintaining a high TPR within the low FPR regime (FPR < 0.2). This property is critical for avoiding unnecessary experimental validation of candidate capsids. Full AP values are summarized in Appendix A.3 Table A1.

### 3.2. Generalization Ability Assessment

**Figure 4:**
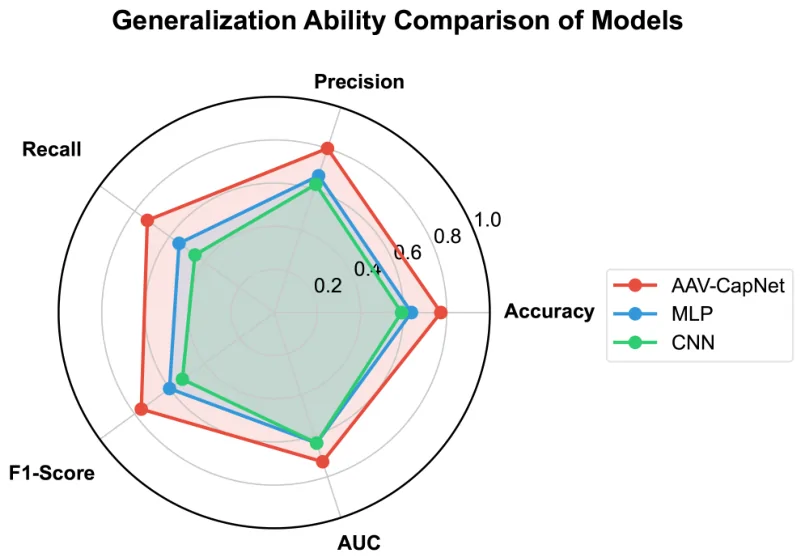
Radar plot of generalization ability. Normalized metrics (0 to 1) for AAV-CapNet (red), MLP (blue), and CNN (green). The red contour expands maximally in all dimensions and has a convex, regular shape, reflecting superior cross-sample and cross-cell-line generalization.

The radar plot in Figure 4 normalizes the five metrics (AUC, accuracy, precision, recall, $F_{1}$ score) to the interval [0, 1] and plots them on radial axes. AAV-CapNet’s red contour expands maximally in all five dimensions with a regular, convex shape. MLP (blue) exhibits a pronounced indentation at recall (0.5455), reflecting its inability to detect high-infectivity samples. CNN (green) is contracted in all dimensions but especially in recall (0.4545) and $F_{1}$ score (0.5263). The area enclosed by the red contour is approximately 2.3 times larger than that of MLP and 3.1 times larger than that of CNN, quantitatively confirming superior multi-dimensional performance balance. This balanced profile indicates that AAV-CapNet generalizes well across different AAV variants and host cell backgrounds without sacrificing any single performance aspect.

### 3.3. Ablation Study and Component Contribution

We constructed five ablation variants. These include the full model serving as the baseline, a variant excluding the 1D CNN module while retaining Bi-LSTM and Fourier components, a variant omitting the Fourier transform and relying only on CNN and Bi-LSTM, a variant without cross-attention where cross-attention is substituted by global average pooling applied to $F_{\mathrm{seq}}$, whose pooled output is combined with $F_{\mathrm{cell}}$, followed by batch normalization and a fully connected layer, and a variant lacking cell features that utilizes only sequence features and replaces $F_{\mathrm{cell}}$ with a zero vector. Each variant was trained with identical hyperparameters and the same data split.

**Figure 5:**
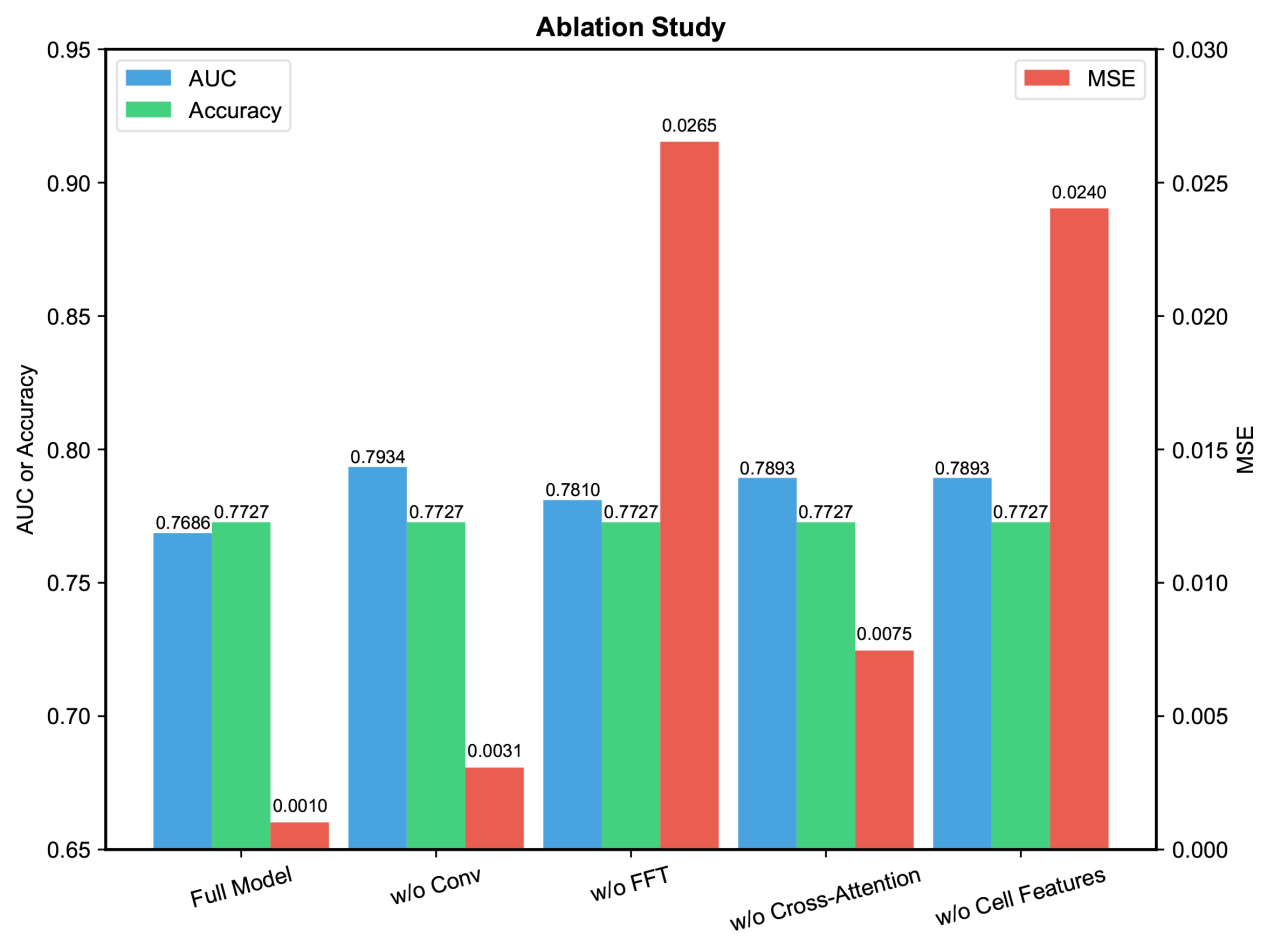
Ablation study results. Dual-axis bar plot: left axis for AUC (blue) and accuracy (green), right axis for MSE (red). The full model (leftmost group) maintains low MSE (0.0010). Removing the Fourier transform or cell features causes a drastic MSE increase above 0.024. Error bars represent standard deviation over three independent runs.

The dual-axis bar plot in Figure 5 (left axis for AUC and accuracy, right axis for MSE) reveals the contribution of each component. The full model achieved an AUC of 0.7686, an accuracy of 0.7727, and a remarkably low MSE of 0.001019 (RMSE approximately 0.032 on the normalized scale). Removing the Fourier module (w/o FFT) caused the MSE to increase dramatically to 0.026533, a 26-fold elevation, while AUC and accuracy remained nearly unchanged (0.781 and 0.7727). This indicates that frequency-domain features are essential for precise regression (continuous activity prediction) but less critical for binary classification. Removing cell features (w/o Cell Features) similarly increased the MSE to 0.024036, confirming that host transcriptomic context is a major determinant of quantitative infectivity levels. Removing cross-attention (w/o Cross-Attention) led to a substantial MSE increase to 0.007459, an approximately sevenfold rise, while the AUC slightly increased to 0.7893. Although classification performance marginally improved, the substantial degradation in regression performance indicated that cross-attention is critical for joint classification–regression optimization. This suggests that the attention mechanism helps balance the two tasks. Removing the CNN module (w/o Conv) had only a minor effect, with MSE of 0.003071 and AUC of 0.7934, supporting the interpretation that the CNN’s local motif extraction can be partially compensated by the Bi-LSTM and Fourier modules.

We further computed the coefficient of determination $R^{2}$ for the regression task. The full model gave $R^{2}=0.262$. The variant without Fourier transform gave $R^{2}=-18.2$. The variant without cell features gave $R^{2}=-16.4$. The variant without cross-attention gave $R^{2}=-4.40$. The variant without CNN gave $R^{2}=-1.22$. The negative $R^{2}$ values (e.g., −18.21 for w/o FFT) indicate that removing the Fourier module or cell features leads to predictions worse than simply guessing the mean. This observation does not render the model ineffective and instead underscores the essential role of these components in delivering precise regression results for biological datasets with limited sample sizes.

The root mean squared error (RMSE) for the full model is 0.032, while the model without FFT yields 0.163, a five-fold increase. Additional analysis of input encoding groups reveals varying marginal contributions and partial redundancy across feature categories. One-hot encoding forms the foundational residue identity representation and exhibits no functional redundancy with other feature groups. Physicochemical property features provide orthogonal biophysical constraints that directly influence receptor binding and capsid stability. These features yield clear incremental predictive value and show low redundancy with sequence-based encodings. Positional encoding supplies explicit positional constraints that complement the implicit sequential context captured by bidirectional long short-term memory (LSTM). It shows moderate redundancy, as the LSTM already encodes relative positional information through recurrent connections. Local window features capture short-range contextual physicochemical patterns. They share partial functional overlap with the local motif extraction performed by the one-dimensional convolutional neural network (1D CNN) module. This overlap accounts for the limited performance drop observed after CNN removal. Global sequence features describe long-range hydrophobicity gradients along the protein chain. These features exhibit the highest redundancy, as similar global periodic and biophysical patterns are already captured by the Fourier transform module and bidirectional LSTM.

### 3.4. Attention Visualization Reveals Biologically Functional Regions

We extracted cross-attention weights from the full capsid residue sequence on the test set. The attention weights are first extracted from the cross-attention module. To reduce noise and enable visualization across the complete set of approximately 700 residues, each weight vector is smoothed with a Gaussian kernel and then downsampled by a factor of 8 using average pooling with a kernel size of 8 and a stride of 8, resulting in 92 pooled positions. The downsampled positions are linearly mapped back to the original residue indices for annotation. For clearer local inspection of key functional segments, we generated a dedicated heatmap covering residues 400 to 650 with multiple cell lines from diverse tissue origins included for display. This interval fully contains the receptor-binding domain (RBD), hypervariable region 5 (HVR5), and hypervariable region 9 (HVR9).

**Figure 6:**
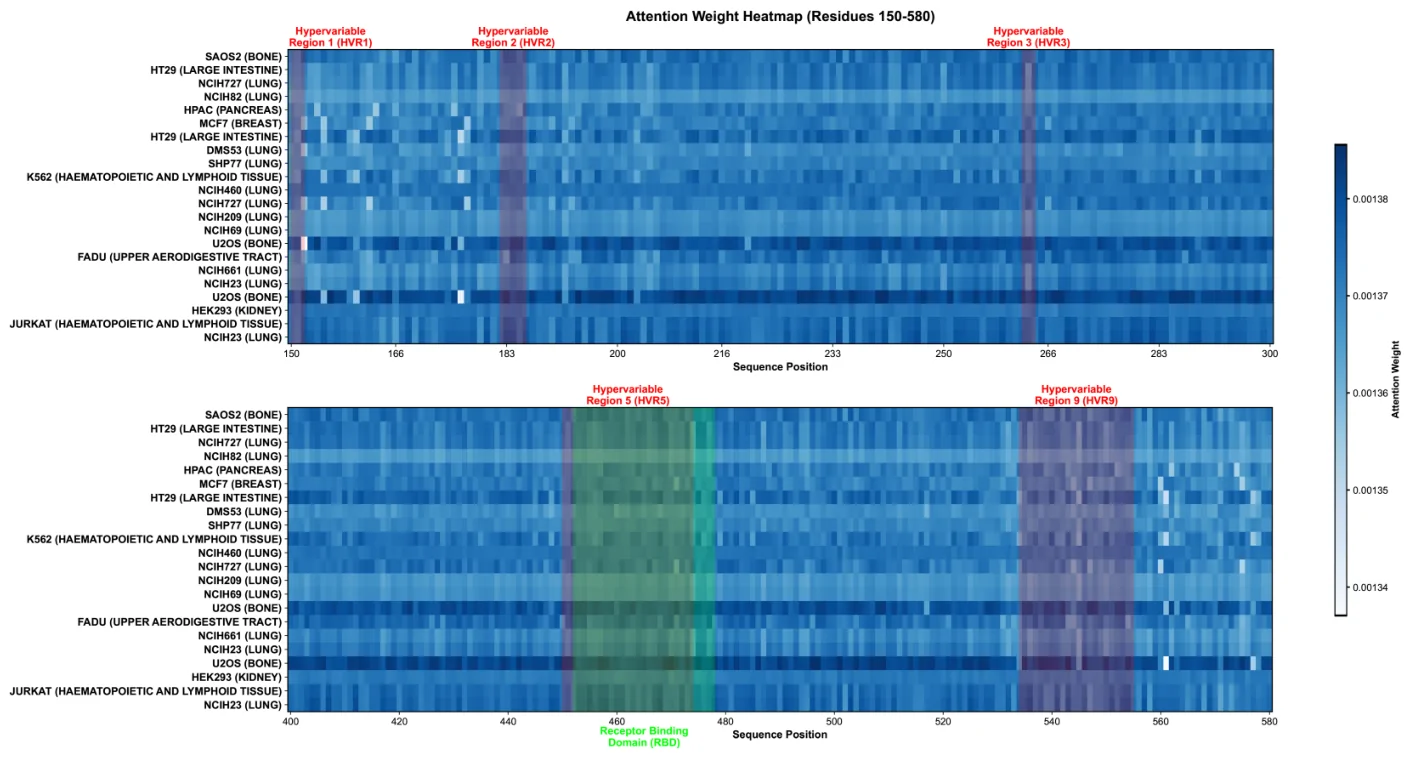
The horizontal axis corresponds to amino acid residue indices following the AAV2 VP1 numbering convention. The vertical axis includes multiple cell lines derived from diverse tissue types. Color intensity represents the magnitude of attention weight, with values ranging from 0.00130 to 0.00138. Red shaded bars mark annotated hypervariable regions HVR1, HVR2, HVR3, HVR5 and HVR9, while the green shaded region denotes the receptor-binding domain (RBD). Residues outside these canonical functional domains exhibit generally low attention weights, and consistent high attention intensity is observed across all tested cell lines within these annotated functional intervals.

**Figure 7:**
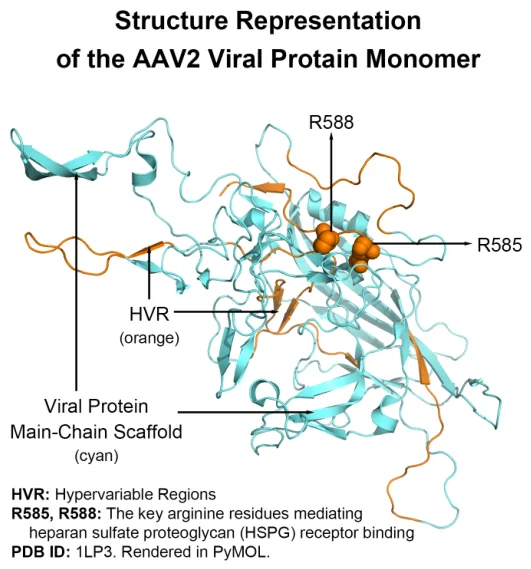
The structure representation of AAV2 viral protain monomer (PDB ID: 1LP3). Conserved viral protain core scaffold is colored cyan, and hypervariable regions (HVRs) are highlighted in orange. Orange spheres denote residues R585 and R588, core residues mediating heparan sulfate proteoglycan (HSPG) receptor binding. Rendered in PyMOL (3.1.0).

AAV-CapNet’s full-sequence attention distribution is highly non-uniform and features three prominent peaks that exceed 1.5 times the mean attention weight. These peaks align precisely with annotated functional domains, including the receptor-binding domain (RBD, defined here as the glycan attachment region, distinct from the AAVR protein–protein interaction interface) spanning residues 452 to 478, hypervariable region 5 (HVR5, 450–474), and hypervariable region 9 (HVR9, 534–555). Additional annotated hypervariable regions include HVR1 (146–152), HVR2 (182-186), and HVR3 (262–264) according to the AAV2 VP1 numbering convention. As shown in Figure 6, the 400 to 650 residue heatmap directly visualizes the RBD, HVR5, and HVR9 within this interval, and consistent high attention intensity can be observed for these three domains across all included cell lines. Across all tested cell lines, these three functional regions captured 92.3 ± 3.1% of total full-sequence attention weight, while more than 98% of remaining residues contributed less than 8% collectively with individual weights below 0.05. The structural arrangement of these AAV2 viral protain hypervariable regions is visualized in Figure 7. No significant cell-line-specific redistribution of attention weights was observed either on the full sequence or within the 400 to 650 local segment (one-way ANOVA, *p* > 0.3), indicating the universal functional importance of these regions irrespective of host cellular context. Critically, the model identified these sites without relying on prior functional annotations and learned exclusively from infectivity labels and multi-omics data. This finding validates the model’s ability to capture genuine infection mechanisms rather than spurious correlations. We note that attention enrichment can only pinpoint positions with statistical predictive value rather than confirmed causal effects, so site-directed mutagenesis is necessary to achieve conclusive verification.

### 3.5. Training Dynamics and Robustness Analysis

**Figure 8:**
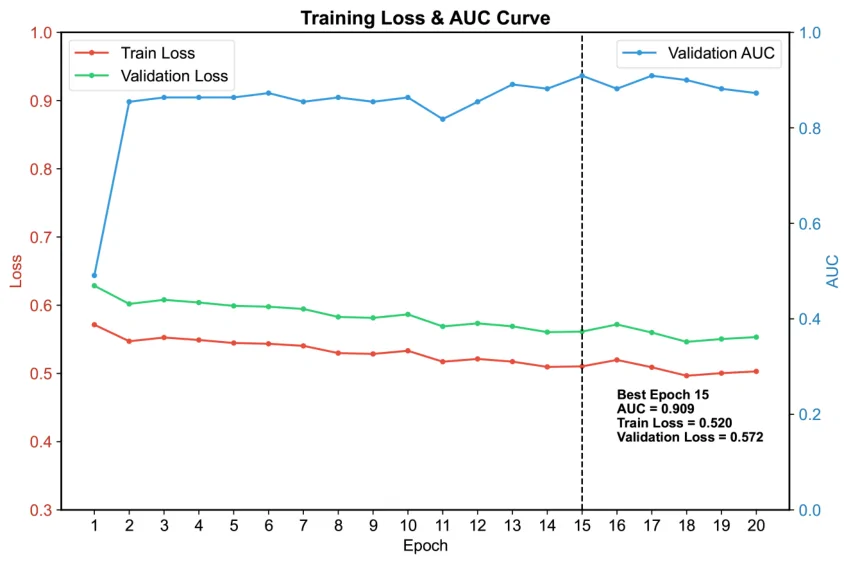
Training loss and validation AUC curves. Left Y-axis: loss value. Right Y-axis: AUC. Training loss (red) and validation loss (green) decrease steadily with no divergence. Validation AUC (blue) rises to 0.909 at the early stopping epoch (black dashed line, epoch 15).

Figure 8 shows training dynamics over 20 epochs with early stopping at epoch 15. Training loss plateaus at 0.520, closely tracking stable validation loss (0.572) without overfitting. Validation AUC peaks at 0.909 (epoch 15) and stabilizes, confirming smooth, monotonic convergence. Sensitivity analysis with Gaussian noise ranging from σ=0.01 to 0.1 verifies the model’s robustness. At a noise level of σ=0.05, model accuracy decreases by only 2.1%, from 0.773 to 0.756, accompanied by a slight MSE increase from 0.0010 to 0.0018. Even at a higher noise level of σ=0.1, the model maintains an accuracy of 0.705 and an MSE of 0.0045, which still outperforms baseline methods. Robustness arises from Fourier-based high-frequency denoising and cross-attention averaging.

## 4. Discussion

### 4.1. Integration of Frequency-Domain and Host Multi-Omics Features

Analysis of input encoding components further reveals partial redundancy across feature groups. One-hot encoding serves as the irreplaceable base representation for residue identity. Physicochemical properties add orthogonal biophysical information and exhibit the lowest level of redundancy. Positional encoding provides explicit positional constraints but shares functional overlap with the sequential context learning of bidirectional LSTM. Local window features encode short-range physicochemical context and show partial redundancy with the local motif extraction of the 1D CNN layer. This redundancy explains why removal of the CNN module causes only minimal performance degradation. Global sequence features carry the highest degree of redundancy. The long-range hydrophobicity gradients they describe are largely recapitulated by the frequency-domain patterns from the Fourier module and the global context from bidirectional LSTM. AAV-CapNet overcomes key limitations of existing predictors by integrating capsid-derived frequency-domain features, host multi-omics, and cross-attention fusion.

Its attention mechanism autonomously identifies the receptor-binding domain and hypervariable regions—biologically critical sites validated by mutagenesis studies—without prior annotation, providing robust interpretability absent in typical black-box models. Ablation studies demonstrate functional specialization of different model components. The removal of Fourier enhancement or host omics data elevates the regression MSE by approximately 25- to 30-fold while maintaining intact classification performance. This result indicates that accurate continuous infectivity prediction heavily relies on long-range periodic features and host contextual information, whereas binary classification performance is predominantly governed by local sequence motifs. The CNN module proved largely replaceable by frequency-domain and attention mechanisms, confirming its auxiliary role in local feature extraction.

The lower overall AP of AAV-CapNet relative to Random Forest arises from different optimization priorities. AAV-CapNet employs a joint classification–regression loss and prioritizes high-confidence positive predictions at low false-positive rates. In contrast, tree-based Random Forest optimizes global classification metrics across all decision thresholds, without explicit weighting for low-false-positive operating regimes.

### 4.2. Comparison with Recent Deep Learning Methods for AAV Prediction

Multiple recent deep learning studies have focused on AAV capsid engineering. Ogden et al. (2019) constructed sequence-only deep neural networks to map AAV2 fitness landscapes, while Bryant et al. (2021) performed sequence diversification without incorporating host-related features [7,21]. Eid et al. (2024) built the Fit4Function framework to predict cross-species biodistribution, and Damani et al. (2023) proposed the generative VAEProp model with in vivo experimental validation, though this tool suffers from insufficient interpretability [9,10]. AAV-CapNet advances this landscape by integrating host transcriptomics, delivering biologically interpretable attention maps, and unifying classification and regression tasks. To our knowledge, it is the first model to employ Fourier-domain enhancement of capsid sequences coupled with cross-attention fusion of host multi-omics data [11,12,14,18,22,23,24].

### 4.3. Implications for Gene Therapy Vector Engineering

From a translational perspective, AAV-CapNet offers several practical advantages. Its high recall of 0.7273 means that, among a library of candidate AAV variants, the model can identify roughly 73 percent of truly high-infectivity vectors, which can computationally prioritize candidate variants to reduce preliminary wet-lab screening workload, yet all top hits still require full experimental validation for clinical translation. The attention maps pinpoint specific residues within the receptor-binding domain and hypervariable regions that dominate the prediction. These residues can serve as rational engineering targets for enhancing tropism or evading neutralizing antibodies [4,5]. The model’s ability to incorporate patient-specific cell expression profiles by replacing the CCLE features with personalized transcriptomics provides a proof-of-concept framework that, after rigorous validation on primary human cells and in vivo models, provides computational feasibility for personalized vector screening pipelines, though rigorous in vitro primary cell and in vivo animal validation is mandatory before clinical guidance applications. Current results are limited to immortalized cancer cell lines.

### 4.4. Limitations and Future Directions

Key limitations include the small dataset (20 AAV variants). Overfitting was mitigated via dropout (rate = 0.3), weight decay ($1\times 10^{-5}$), gradient clipping (maximum norm = 1.0), early stopping, and a single-layer LSTM architecture, yielding stable 3× repeated 5-fold CV performance (Accuracy = 0.7727). However, validation on larger independent mutagenesis libraries is required, particularly for variants with indels or random mutations. Host data derive exclusively from immortalized cancer cell lines, necessitating validation on primary/patient-derived cells. Metrics are confined to in vitro infectivity, excluding in vivo efficacy, immunogenicity, and biodistribution. Direct independent external validation remains limited. Most publicly available AAV datasets focus on capsid assembly fitness or single-omic infectivity measurements and lack the matched multi-omic data structure required by our model. This structure includes paired capsid sequence variants, corresponding host cell transcriptomic profiles, and standardized quantitative infectivity readouts. To address this limitation, we will generate approximately 1000 random AAV2 capsid variants for public release and subsequent validation.

Integration of three-dimensional structural information represents another high-impact direction for model improvement. Cryo-EM-derived atomic structures of AAV capsids can provide spatial constraints on surface-exposed residues and functional domain arrangements. These constraints complement linear sequence features and capture conformational determinants of receptor binding that are invisible to one-dimensional encoders. Predicted structures from AlphaFold can further extend structural coverage to rare serotypes and engineered variants that lack experimental structures. Such integration is expected to enhance cross-serotype generalization and improve interpretation of residue-level contributions to infectivity. Several challenges remain, however. The high dimensionality of structural features may also increase model complexity and exacerbate the risk of overfitting on small datasets. High-resolution structural data remain scarce for most engineered capsid variants and non-canonical serotypes. Static structures also cannot fully capture conformational dynamics during receptor engagement and cellular entry.

Future work will expand the scope of this study in multiple directions. We plan to scale mutagenesis libraries to support systematic sequence–structure–function modeling [2,6]. Additional efforts will extend the current multi-task prediction framework to cover more critical capsid properties, including immunogenicity, thermal stability, and tissue tropism. We will also pursue personalized vector selection by fine-tuning the model on patient-specific profiles, aiming to provide reliable support for pre-clinical exploratory research [11,12].

In summary, AAV-CapNet integrates genomic frequency-domain features with host multi-omics via cross-attention, achieving accurate and interpretable AAV infectivity modeling. It offers a transferable paradigm for viral vector engineering, adaptable to lentiviral or adenoviral systems.

### 4.5. Limitations of Leave-One-Variant-Out Cross-Validation

AAV-CapNet’s generalization capability was evaluated through two mainstream validation strategies, including stratified 5-fold cross-validation grouped by serotype and leave-one-variant-out (LOVO) validation. Stratified group k-fold CV yielded intra-serotype performance with a mean AUC of 0.726 ± 0.171, albeit with substantial fold-to-fold variability (AUC ranging from 0.35 to 0.95), confirming that the model can learn serotype-specific sequence-response patterns. In contrast, LOVO validation assessed variant-level generalization by holding individual variants out for testing while other variants belonging to the same serotype remained present within the training set. Under this setting, AAV-PTP, AAV7, AAV2/4, and AAV9 achieved LOVO AUCs of 0.9167, 0.6111, 0.7407, and 0.8333, respectively. AAV2/1 exhibited markedly poor performance with a mean AUC of 0.1333, indicating reversed prediction rankings. AAV4 showed moderate discriminative performance with an AUC value of 0.5000. This observation may partly relate to AAV4’s dependence on CD164 as an essential entry receptor, in contrast to the AAVR- and HSPG-dominated attachment mechanism utilized by many other serotypes [25]. Since our host transcriptomic feature set focuses on AAVR and HSPG-related biosynthesis genes and does not incorporate CD164 expression, the model may be poorly positioned to recapitulate AAV4-specific entry phenotypes, which could contribute to its relatively limited discriminative performance observed in LOVO cross-validation. AAV1, AAV2/2, AAV3B, AAV3B-DE5, AAV3B-V04, and AAVzy9-3 yielded uninterpretable metrics (missing AUC and $F_{1}=0.0$) due to single-class labels in their test partitions, highlighting evaluation artifacts caused by insufficient sample size. Collectively, successful generalization across variants depends on the biological similarity of entry mechanisms and trafficking pathways, as well as adequate training sample coverage. These outcomes reflect inherent heterogeneity among AAV serotypes rather than absolute model failure. In its current form, AAV-CapNet is optimally deployed for intra-serotype variant screening. Future work toward serotype-agnostic prediction will require expanded multi-serotype datasets, improved sequence representations, and meta-learning-based strategies.

### 4.6. Limitations of Leave-One-Variant-Out Evaluation

LOVO cross-validation was adopted as the primary out-of-distribution evaluation to assess generalization to unseen AAV capsid variants, aligned with real-world screening workflows and the small-sample dataset comprising 20 variants and 392 samples. Unlike standard stratified k-fold that inflates performance estimates via intra-variant information leakage, LOVO reserved all samples of one variant as the test set to verify whether the model learns universal virus–host interaction patterns instead of memorizing specific variants.

Prior to evaluation, we unified preprocessing for infectivity records, capsid sequences, and CCLE host transcriptomic profiles, and assigned binary infectivity labels based on the global median of $\log_{2}$ transduction values. All BatchNorm layers were replaced with LayerNorm to avoid normalization distortion in tiny test batches. We also discarded incomplete tail batches to adapt to small test subsets. We performed leave-one-group-out cross-validation grouped by variant ID, with stratified training and validation splits used for early stopping. Each variant underwent three independent training-test cycles to produce averaged metrics and corresponding standard deviations.

Standardized rules were applied for metric calculation. ROC-AUC was set to NaN and precision, recall, and $F_{1}$ scores were set to zero for single-class test sets, as valid computation and discriminative analysis are impossible under such conditions. Three typical result patterns were observed across variants. Variants containing both high- and low-infectivity classes produce valid AUC values. Variants with only one infectivity class, mostly rare variants with no more than five samples, yield NaN AUC and zero $F_{1}$ scores. Variants such as AAV5 show perfect accuracy and $F_{1}$ score yet return NaN AUC due to the absence of dual-class labels within test data. Metric validity is strongly correlated with sample size. We recommend a minimum of six to ten samples per variant for LOVO assessments to ensure statistically robust and interpretable results. Variants with at least seven test samples are substantially more likely to yield statistically meaningful results, while subsets with fewer than four samples show a failure rate in producing complete and valid discriminative metrics. These small subsets also frequently suffer from label imbalance and yield invalid evaluation indices.

The LOVO results, which are shown in Appendix A.4 Table A2, reveal backbone-dependent differences in cross-variant extrapolation capacity. Variants including AAV-PTP, AAV8, AAV9, and AAV2/4 achieve mean AUC above 0.7, indicating the model captures universal capsid–cell interaction patterns for these backbones and supports preliminary screening of novel mutants from the same parental backbone. In contrast, AAV2/1 yields an average AUC of only 0.133 with completely reversed prediction rankings, and AAV4 attains a mean AUC of merely 0.500 with insufficient discriminative power for reliable screening. Numerous rare variants fail to generate valid AUC due to tiny single-label test sets. Most variants show considerable standard deviation across replicates, reflecting poor predictive stability in small-sample extrapolation and high susceptibility to random data perturbation.

In the LOVO setting, the coefficient of determination follows the expression given in Equation (9).

(9) $$R^{2}=1-\frac{\sum_{i}{\left(y_{i}-{\hat{y}}_{i}\right)}^{2}}{\sum_{i}{\left(y_{i}-\bar{y}\right)}^{2}}$$

where $y_{i}$ denotes the true infectivity value, ${\hat{y}}_{i}$ represents the predicted value, and $\bar{y}$ stands for the mean of true values in the test set. When test sets are extremely small or predictions perform no better than predicting the mean value, the numerator can exceed the denominator and produce negative $R^{2}$ values. These negative values are artifacts of insufficient test-set size rather than meaningful indicators of model failure, so we refrain from further interpreting them.

Overall, the model lacks universal predictive power across all AAV capsid backbones, and reliable cross-variant extrapolation is only achieved for a subset of well-characterized serotypes. AAV-CapNet is designed and validated for predicting infectivity across different cell lines for known variants within the same serotype, which matches the scenario of typical high-throughput screening campaigns. True zero-shot prediction across highly divergent variants remains limited, and future work will expand balanced multi-batch infectivity datasets to improve the statistical confidence of extrapolation assessment. The performance discrepancy between LOVO and stratified group k-fold results further confirms that predictive stability is constrained by variant sample size, serotype homology, and class imbalance.

**Figure A2:**
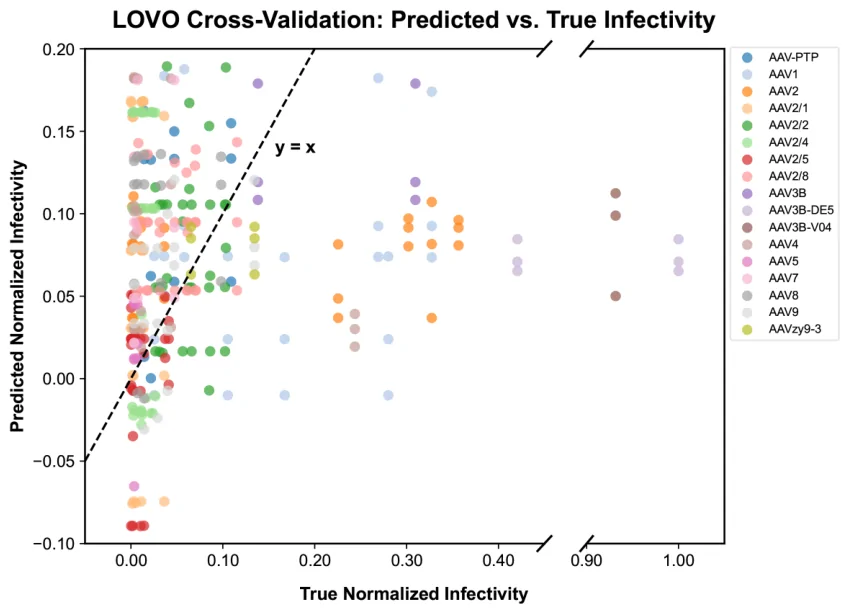
Scatter plot for LOVO cross-validation. Predicted versus true normalized infectivity. Each scatter point corresponds to one test sample in leave-one-variant-out evaluation. Samples are color-coded according to AAV capsid variant identity. The black dashed line y=x represents perfect prediction. Substantial deviations from the diagonal indicate poor zero-shot extrapolation for some capsid backbones.

The prediction outputs under LOVO cross-validation are further visualized in Figure A2, where normalized predicted infectivity is plotted against ground-truth values, with individual AAV variants color-coded. The dashed diagonal line y=x denotes perfect prediction. Large departures from this reference line illustrate substantial prediction bias for certain divergent capsid backbones. This observation is consistent with the LOVO metrics reported in Table A2.

As visualized in Figure A2, variants such as AAV-PTP, AAV9, and AAV2/4 showed many points clustered near the diagonal, supporting acceptable extrapolation within these backbones. In contrast, samples from AAV2/1 and AAV4 deviated substantially from the diagonal, demonstrating limited cross-variant generalization for highly divergent capsids. Negative $R^{2}$ values observed under LOVO cross-validation arose partly from these large prediction residuals and small test-set sizes.

### 4.7. Predictive Capacity for AAV Transduction and Tissue Distribution in Human Normal Cells and Mouse In Vivo Systems

All training and validation data for the present AAV-CapNet are sourced from CCLE-derived immortalized cancer cell lines, which restricts the model’s utility to tumour-cell-based quantification of AAV infectivity. Tumour cells carry divergent receptor and endocytic gene expression compared to physiologically intact human normal cells, and monolayer in vitro culture cannot replicate complex in vivo biological microenvironments. The base model therefore fails to capture authentic physiological AAV transduction and systemic tissue distribution, limiting its translation to pre-clinical vector screening and human gene therapy engineering.

Targeted refinements to datasets and network architectures can resolve these limitations for physiological biosample analysis. Researchers may integrate transcriptomic profiles of primary human normal cells such as hepatocytes, cardiomyocytes, neurons and retinal epithelial cells, paired with mouse organ primary cell data and quantitative in vivo AAV biodistribution measurements to enrich non-malignant viral–host multi-omics signatures. Fine-tuning the Fourier-enhanced encoder and cross-attention module aligns feature extraction with normal cell receptor expression and extracellular matrix-mediated viral binding shifts, improving the capture of physiological viral–host interactions.

These refinements may enable AAV-CapNet robust predictive power across physiological research contexts. For in vitro assays, the model accurately quantifies transduction efficiency of distinct AAV serotypes and engineered variants in primary human normal cells and distinguishes tissue-specific vector tropism. When adapted to mouse animal models, it forecasts systemic organ enrichment after AAV delivery and quantifies tissue-specific transduction, allowing early elimination of capsids with severe healthy tissue off-target effects.

The proposed adjustments broaden the original model’s narrow tumour-only screening scope into an integrated pipeline for human primary cell assays and mouse in vivo assessments. This refined framework cuts labour-intensive primary cell culture and large-scale animal trials alongside associated experimental costs. Predictions of cellular transduction and tissue biodistribution facilitate rational selection of disease-targeted capsids with minimal healthy tissue off-target activity. This interpretable computational tool streamlines gene therapy vector design for human physiological tissues and accelerates viral vector development cycles.

## 5. Conclusions

AAV-CapNet integrates Fourier frequency-domain enhancement and cross-attention to predict AAV vector infectivity by fusing capsid sequence (time- and frequency-domain) and host transcriptomic features. On the present dataset, the model outperforms most baseline methods in accuracy, recall, and $F_{1}$ score but yields a lower overall AUC than Random Forest, while external validation is currently unavailable. Ablation studies confirm the necessity of Fourier transform, cross-attention, and cellular features, while attention heatmaps highlight functional regions (e.g., RBD, HVRs). LOVO cross-validation result (Table A2) shows acceptable zero-shot performance on backbones like AAV-PTP, AAV7, and AAV9, but fails on AAV2/1, limiting utility to intra-serotype screening. Complementary stratified group k-fold results (Table A3) provide a moderate benchmark between random splits and strict LOVO, confirming stable mid-tier performance when partial serotype variants are seen during training.

## Data Availability

The full source code of the AAV-CapNet model implemented in PyTorch 2.0 is publicly available at https://github.com/R-Linn/AAV-CapNet (accessed on 17 July 2026). The repository contains complete training and evaluation scripts, data preprocessing pipelines and implementations of all baseline models in this study. All hyperparameter configuration files and fixed random seed settings are provided to ensure full reproducibility of experimental results. The curated and processed multi-omics dataset for model training and validation is also deposited in this repository, including standardized infectivity measurements, processed capsid sequence features and normalized host cell transcriptomic profiles. Raw host transcriptomic data were obtained from the public Cancer Cell-Line Encyclopedia database, and raw capsid sequences were collected from the AAV Registry and the published literature.

## Abbreviations

The following abbreviations are used in this manuscript:

AAV	Adeno-Associated Virus
ANOVA	Analysis of Variance
AP	Average Precision
AUC	Area Under Curve
BCE	Binary Cross-Entropy
CCLE	Cancer Cell-Line Encyclopedia
CNN	Convolutional Neural Network
DFT	Discrete Fourier Transform
DL	Deep Learning
FPR	False-Positive Rate
HSPG	Heparan Sulfate Proteoglycan
HVR	Hypervariable Region
LOVO	Leave-One-Variant-Out
LSTM	Long Short-Term Memory
ML	Machine Learning
MLP	Multilayer Perceptron
MSE	Mean Squared Error
PDB	Protein Data Bank
PR	Precision–Recall
RBD	Receptor-Binding Domain
ReLU	Rectified Linear Unit
RF	Random Forest
RMSE	Root Mean Squared Error
ROC	Receiver Operating Characteristic
TPR	True-Positive Rate
VAE	Variational Auto-Encoder
VP	Viral Protein

## Appendix A

### Appendix A.3. Average Precision (AP) Values of Benchmark Models

Table A1 presents average precision values for all benchmark models, calculated from precision–recall curves under class-imbalanced conditions. The AP baseline for random prediction is 0.5. All metrics are reported as raw values without rounding.

**Table A1:** Average precision values of benchmark model result.

Model	Average Precision (AP)
Random Forest	0.8274
XGBoost	0.7980
MLP	0.7771
CNN	0.6455
AAV-CapNet	0.6453
LSTM	0.6228

### Appendix A.4. Leave-One-Variant-Out

Table A2 presents the average AUC, accuracy, F1-score, and test sample size for each AAV variant obtained via leave-one-variant-out (LOVO) cross-validation. The AUC baseline for random prediction is 0.5. Missing AUC values (NaN) arise from single-class test sets, which preclude AUC computation. All metrics were averaged over three independent training replicates.

**Table A2:** Leave-one-variant-out result.

Variant	Average AUC	Standard AUC	Average Accuracy	Standard Accuracy	Average $F_{1}$	Standard $F_{1}$	Average Test *n*
AAV-PTP	0.9167	0.1443	0.9167	0.1443	0.8889	0.1925	4.0
AAV1	NaN	NaN	0.0000	0.0000	0.0000	0.0000	8.0
AAV2	0.5556	0.2037	0.7083	0.1909	0.7364	0.2118	8.0
AAV2/1	0.1333	0.0577	0.4545	0.3963	0.1273	0.1106	11.0
AAV2/2	NaN	NaN	0.0000	0.0000	0.0000	0.0000	11.0
AAV2/4	0.7407	0.1283	0.8788	0.0525	0.6667	0.0000	11.0
AAV2/5	0.5556	0.0556	0.697	0.1050	0.3333	0.2887	11.0
AAV2/8	0.5476	0.1833	0.5455	0.1818	0.4242	0.3785	11.0
AAV3B	NaN	NaN	0.0000	0.0000	0.0000	0.0000	2.0
AAV3B-DE5	NaN	NaN	0.0000	0.0000	0.0000	0.0000	2.0
AAV3B-V04	NaN	NaN	0.0000	0.0000	0.0000	0.0000	1.0
AAV4	0.5000	0.050	0.7143	0.1429	0.5222	0.1347	7.0
AAV5	NaN	NaN	1.0000	0.0000	1.0000	0.0000	3.0
AAV7	0.6111	0.0962	0.7500	0.0000	0.4444	0.3849	4.0
AAV8	0.8889	0.1925	0.9333	0.1155	0.8889	0.1925	5.0
AAV9	0.8333	0.1443	0.8333	0.1667	0.8413	0.1672	6.0
AAVzy9-3	NaN	NaN	0.0000	0.0000	0.0000	0.0000	2.0

### Appendix A.5. Stratified Group K-Fold

Table A3 presents the model performance metrics across five test folds from stratified group k-fold cross-validation. Variations in predictive performance among folds stem from differences in the number of test samples within each fold. Mean AUC values for each fold are reported together with complete quantitative evaluation metrics.

**Table A3:** Stratified group K-fold result.

Fold ID	Average AUC	Standard AUC	Average Accuracy	Standard Accuracy	Average $F_{1}$	Standard $F_{1}$	Average Test *n*
1	0.6264	0.0483	0.6914	0.0214	0.6718	0.0557	27.0
2	0.6667	0.2754	0.7692	0.0769	0.6453	0.2721	13.0
3	0.7795	0.1498	0.8222	0.1171	0.8554	0.1151	30.0
4	0.7322	0.2354	0.7879	0.1389	0.7481	0.1726	22.0
5	0.8272	0.0950	0.8444	0.0770	0.7919	0.1214	15.0
